# Supplementary material for: High rate of high-risk human papillomavirus among benign and breast cancer patients in Ethiopia
Source: PLoS One. 2024 Mar 20;19(3):e0298583. doi: 10.1371/journal.pone.0298583 (PMC10954173; doi:10.1371/journal.pone.0298583)
Supplement: Attachment — Submitted filename: Response to Reviewers.docx [file pone.0298583.s001.docx]

**The comments of editors and reviewers' comments were constructive and valuable in enhancing the manuscript. We thank all the editors and reviewers for their crucial comments and suggestions. We reflected on a few comments here and all the necessary changes were incorporated into the manuscript in a clean version and with track changes.**

**Reviewer #1:**

1-The Introduction was well described.

**Response:** Thank you for your supportive comments

2-The Materials and methods. The study was not well-designed. Since the results are controversial.

The authors have reported that 14/66(20.6%) cases were positive for HPV while 16/54(29.6%) control was positive for HPV high-risk groups or low-risk groups.

**Response:** Thank you for your comment, this result is for both high-risk and low-risk HPV, and we have included it in the updated version (lines 35, ,306)

It seems some problems in sample collection or some technical problems have taken place in malignancy tumors and even among the control case (benign) detections reports by pathologists.

**Response:** Our pathologists examined all of the slides and made classification according to standard diagnostic procedures to determine whether the breast tumor was benign or malignant (They used Robbins Basic Pathology as a reference). The benign breast tumors included in this study were fibroadenoma and fibrocystic changes.

3- Such work requires detection and expression of HPV 16 E6 mRNA, HPV 31 E6 mRNA, HPV 42 E6 mRNA, HPV 44 E6 mRNA, HPV 45 E6 mRNA, HPV 59 E6 mRNA, and HPV 16 E7 mRNA, HPV 31 E7 mRNA, HPV 42 E7 mRNA, HPV 44 E7 mRNA, HPV 45 E7 mRNA, HPV 59 E7 mRNA among the malignancy and beings tumors’ samples.
4- Therefore, the results and discussion cannot be justified.
5-No novelty.

**Response:** Thank you, **t**his feedback is valuable. In this study, to our knowledge, we found an association with HPV and ER for the first time (Line 518, 600). We understand detection and expression of E6 mRNA and E7 mRNA among HPV-positive cases is important, and we will consider it for our future study.

**Reviewer #2:** This manuscript tackles a controversial area of research that has not conclusively established a causal link between breast cancer and HPV infection. The authors investigated a well-established fact that HPV infection occurs in breast cancer tissue. They examined the extent of HPV infection in Ethiopian cases -though whether female or male cases were chosen is not described. Their novel finding is the differentially high distribution of HPV infection in ER+ tumors-

**Response:** Thank you for your encouraging comments, we have only chosen females and are now included in the method section line 175.

Would the authors please address the following queries?

INTRODUCTION
1) In the introduction please provide your rationale for selecting HPV over the other viruses mentioned for exploration in your Ethiopian sample- 2) I suggest that from the discussion section, lines 191 starting from The existence of a causal link....202 should be put into the introduction section as part of the rationale for studying HPV and its potential etiological link to breast cancer development.

**Response:** Thank you for your comments, we added and moved your suggested discussion section into the introduction as part of the rationale, lines 84-87. Another rationale is also indicated in line 87, 124,125.

METHODS SECTION

1) Please explain your rationale for sample selection there is no sample size calculation to justify your sample size selection -how can you be sure that your sample size is sufficient to give meaningful results- particularly for the distribution of receptor subtyping -is this representative of population receptor subtype distributions?

**Response:** Your comments are appreciated; this study was a pilot exploratory. We have calculated the sample size using Epi-info version 7.2.1.0, a statistical software indicated in lines 141-147

In addition, your regression analysis is not described in the analysis section of your methods -please correct this.

**Response:** Your comments are appreciated, based on your comments lines 293 to 296 have been updated.

a) 85% of the sample selected were from very young patients- is this the typical age distribution of breast cancer among the Ethiopian population?

**Response:** Thank you for your comment, Ethiopian breast cancer patients fell within the younger age group category in general(indicated in lines 315, 316, 335 and 336

b) Stage at diagnosis is not presented in Table 2 and should be given to give an accurate representation of size, nodal involvement, and metastatic invasion- what proportion of patients had stage 3 &4 disease?

**Response:** Thank you for your comment, based on your comment, Table 2 has been updated.

2) Table 2 confuses me - your heading says associated factors for HPV infection among BC and non-malignant breast cases -yet tumor size, lymph nodes grade receptor subtyping is only for cases- I feel it would be much clearer if you had major column headings as CASES and CONTROLS and TOTALS with subheadings under each as HPV+ HPV-. Naturally describing the tumor features would be left blank for controls -but as it stands your table is confusing- also HER2 should be expressed as negative, equivocal, and positive.
   3) For Table 3 you have not defined COR and AOR -is COR crude odds ratio and AOR adjusted odds ratio -if so then include these definitions in your footer

**Response:** Thank you for your crucial comments; Table 2 was unclear. To avoid this confusion, we choose to write the age group data in text form. As a result, Table 2 now only includes breast cancer cases. We included the HER-2 equivocal cases in the chi-square test but excluded them from the regression analysis because we only used binary independent variables.

In the footer of Table 3, we have also included definitions for the COR crude odds ratio and AOR adjusted odds ratio.

DISCUSSION SECTION

1)Please first summarise your major findings in the first paragraph

**Response:** Thank you for your comment, as per your comments,  included our major finding in the first paragraph of the discussion.

2) Move lines 191-202 to the introduction section as part of the rationale for selecting HPV from the other viruses to study

**Response:** Thank you for your comment, as per your comments,  we moved it to the introduction line 84-87.

3) Line 212 adds the word "development"

**Response:** Thank you for your comments, we have included them in line 586.

4) line 214 clarifies what you mean by "long-term breast cancer" -is this advanced breast cancer?---

**Response:** To avoid reader confusion we have removed this term indicated in line 589.

**Reviewer #3:** The article explores an intriguing topic. Strengthening the writing style could potentially elevate the quality of the work, providing a more polished and engaging reading experience for the audience-

Please improve the whole manuscript writing in a way of methodology, table result display, analysis, and discussion.

**Response:** Thank you for your encouraging comments, we improve and strengthen the writing style.

**Reviewer #4:** Congratulations to the authors for undertaking this research project which aims to add to the body of knowledge concerning Breast cancer & HPV, which remains one of the latest controversial issues. Some concerns I have with the manuscripts:

1. The study aimed to investigate the magnitude of HPV infection among breast cancer in Ethiopia, however, the conclusion speaks of ' ....no proof of a link between HPV and cancer '. ' How, did they determine that there was no link or association? How did they get to this conclusion? (Sentence 181 & conclusion 224)

**Response:** Comment accepted and included, and this sentence is now modified 518 and 532 and throughout the manuscript.

2. Sentence 190-191: As above, the author cannot make the implication that implication solely on the fact that HPV % was higher in benign than cancerous lesions. Moreover, instead of 'imply' perhaps the author may write ' which may suggest'

**Response:** Thank you for your comment, the comment is accepted and included in line 540

3. Sentence 184: Needs paraphrasing

**Response:** Thank you for your comment, done, line 534

4. Sentence 185: No reference-

**Response:** This is the reference used for “The role of integration in oncogenic progression of HPV-associated cancers” for this sentence, which is similar to the below sentence. But to avoid confusion we have added the same reference in both sentences.

5. Sentence 188:.......' documented' instead of 'indicated'-

**Response:** Comment accepted and included in line 538

Sentence 201-202: '...does not provide evidence to support HPV's involvement in the development & progression of breast cancer'- This was not determined in this study.

**Response:** Thank you for your comment, the comment is accepted and included and this sentence is now modified in the whole manuscript
