# Supplementary material for: High rate of high-risk human papillomavirus among benign and breast cancer patients in Ethiopia
Source: PLoS One. 2024 Mar 20;19(3):e0298583. doi: 10.1371/journal.pone.0298583 (PMC10954173; doi:10.1371/journal.pone.0298583)
Supplement: Attachment — Submitted filename: Response to Reviewers.docx [file pone.0298583.s002.docx]

**The comments of both editors were constructive and valuable in enhancing the manuscript. We thank all the editors for their crucial comments and suggestions. We reflected on a few comments here and all the necessary changes were incorporated into the manuscript in a clean version and with track changes.**

**Academic Editor:** Please review your reference list to ensure that it is complete and correct. If you have cited papers that have been retracted, please include the rationale for doing so in the manuscript text, or remove these references and replace them with relevant current references. Any changes to the reference list should be mentioned in the rebuttal letter that accompanies your revised manuscript. If you need to cite a retracted article, indicate the article’s retracted status in the References list and also include a citation and full reference for the retraction notice.

**Response:** all references have been updated. However, we have removed reference 41 (OHBA, K., CHONG, P. P. & YAMAMOTO, N. 2017. Role of Human Papillomavirus (HPV), Estrogen and Apobec3B Axis in Breast Cancer Initiation **Line 216**) as it has now been retracted.

**Additional Editor Comments:**

Dear Authors,

Thank you for your submission to Plos One.
I think most of the concerns are explained and fully revised, but I have a little concern that HPV infections are both high-risk and low-risk types.
Could you divide this population into high and low-risk HPV-type groups and look at the differences?

**Response:** This feedback is really helpful, and we have amended it in Table 1 . Line 156. Because there are fewer than ten cases in the low-risk HPV groups, regression, and chi-square analysis cannot be performed to further examine it. In most circumstances, group variables of less than 10 should not be compared for statistical regression and chi-square analysis.
